# Maximizing mRNA vaccine production with Bayesian optimization

**DOI:** 10.1002/bit.28216

**Published:** 2022-09-05

**Authors:** Sara Sousa Rosa, Davide Nunes, Luis Antunes, Duarte M. F. Prazeres, Marco P. C. Marques, Ana M. Azevedo

**Affiliations:** ^1^ Department of Bioengineering, iBB—Institute for Bioengineering and Biosciences, Instituto Superior Técnico Universidade de Lisboa Lisboa Portugal; ^2^ Associate Laboratory i4HB—Institute for Health and Bioeconomy, Instituto Superior Técnico Universidade de Lisboa Lisboa Portugal; ^3^ LASIGE Faculdade de Ciências da Universidade de Lisboa Lisboa Portugal; ^4^ Department of Biochemical Engineering University College London London UK

**Keywords:** Bayesian optimization, in vitro transcription, machine learning, mRNA, vaccines

## Abstract

Messenger RNA (mRNA) vaccines are a new alternative to conventional vaccines with a prominent role in infectious disease control. These vaccines are produced in in vitro transcription (IVT) reactions, catalyzed by RNA polymerase in cascade reactions. To ensure an efficient and cost‐effective manufacturing process, essential for a large‐scale production and effective vaccine supply chain, the IVT reaction needs to be optimized. IVT is a complex reaction that contains a large number of variables that can affect its outcome. Traditional optimization methods rely on classic Design of Experiments methods, which are time‐consuming and can present human bias or based on simplified assumptions. In this contribution, we propose the use of Machine Learning approaches to perform a data‐driven optimization of an mRNA IVT reaction. A Bayesian optimization method and model interpretability techniques were used to automate experiment design, providing a feedback loop. IVT reaction conditions were found under 60 optimization runs that produced 12 g · L^−1^ in solely 2 h. The results obtained outperform published industry standards and data reported in literature in terms of both achievable reaction yield and reduction of production time. Furthermore, this shows the potential of Bayesian optimization as a cost‐effective optimization tool within (bio)chemical applications.

## Graphical abstract

mRNA, a forefront technology vaccination field, is produced in an in vitro transcription (IVT) reaction catalyzed by RNA polymerase. In this work, a machine learning approach, in particular, Bayesian optimization, was used as a form of incremental adaptive design‐of‐experiments (DoE) to maximise mRNA production. Optimal reaction conditions (with a production of 12 gmRNA.L‐1 in two‐hours) were found in 60 runs, outperforming published industry standards. These results show the potential of Bayesian optimization as a cost‐effective optimization tool for (bio)chemical applications.

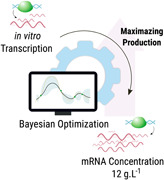

## 1 INTRODUCTION

Delivering vaccines in a short time is key to control disease outbreaks, as recently shown during the Covid‐19 pandemic. However, this is a demanding task, since traditional vaccine manufacturing is complex, time‐consuming, and lacks the flexibility required for a timely and global response (Hayman et al., 2021; Hosangadi et al., 2020). During the Covid‐19 pandemic event, messenger RNA (mRNA) technology allowed for the delivery of an approved vaccine in a record‐breaking time of less than 1 year (Ball, 2020). This success can be attributed to the inherent flexibility and precision of mRNA vaccines, as the same vaccine backbone can be used for multiple targets, and only the gene of interest is expressed. Additionally, mRNA vaccines can be manufactured in a standardized manner, allowing for the production of different vaccine targets with the same platform (Rosa et al., 2021). Nonetheless, global demand has peaked for COVID‐19 vaccines and manufacturers are struggling with the supply chain (Wouters et al., 2021). Scaling up the manufacturing process can be limited, as a result of constraints in materials and equipment availability, and lack of knowledge of the manufacturing process itself (Mishra et al., 2022). Enabling on‐demand vaccine production requires an efficient and cost‐effective manufacturing process that makes optimal use of existing resources.

mRNA vaccines are produced in a cell‐free system that in vitro transcribes the desired DNA template into a mRNA molecule using a RNA polymerase as catalyst and nucleoside triphosphates (NTPs) as substrate. Other reaction components (e.g., magnesium, RNAse inhibitors, and inorganic pyrophosphatase) are added to the reaction medium, which is performed under controlled pH and temperature conditions (Chamberlin, 1982; Geall et al., 2013). The reaction delivers $g_{\mathrm{Product}}\;{L_{\mathrm{Reaction}\;\mathrm{Medium}}}^{-1}$, in a matter of hours, typically 2–5 g .L^−1^ (Bancel et al., 2016; Henderson et al., 2021; Wochner et al., 2021). The fine balance between reaction components and conditions will dictate the reaction outcome, that is, the quantity and quality of mRNA produced. The large number of variables in an in vitro transcription (IVT) reaction can constitute an optimization challenge and a critical problem for Design of Experiment (DoE) methods traditionally employed for reaction optimization (van de Berg et al., 2021). These conventional DoE methodologies can potentially introduce human bias in parameter factor selection and often imply oversimplified assumptions about the parameter relationships. Furthermore, typical DoE approaches can be time and cost prohibitive (e.g., for reactions that require 12 parameters to be evaluated with each parameter having 3 levels to be considered, typically a full factorial design requires over $5\times 10^{5}\;\left(3^{12}\right)$ experimental runs to be performed). Routinely, to reduce the number of optimization runs, some variables are kept constant. These fixed values frequently come from the operator's intuition or from data insights from existing literature, often depicting similarly constrained settings (Carlson & Carlson, 2005). Therefore, new methods are necessary to overcome the traditional limitations of DoE approaches. We therefore propose Bayesian optimization as a methodology that allows for a scientist‐in‐the‐middle approach (Antunes et al., 2007), where domain knowledge (e.g., parameter constraints) complemented with model analysis/explanations are used to fine tune the search, in this particular case, for optimal reaction conditions.

Bayesian optimization is an iterative global optimization method suitable for optimization problems in which a maximization of a black‐box objective function over a bounded set of variables is sought (Jones et al., 1998; Močkus 1975). This method is suitable in situations where experimental time and resources become prohibitive to rapidly perform the optimizations. The method lends itself well to where computational approaches can lead to better parameter selection. Fundamentally, Bayesian optimization has two main components: a surrogate model, and an acquisition function. The surrogate model mimics the behavior of the unknown “expensive” function being optimized, while being computationally “cheaper” to evaluate. It provides a prior probability distribution over all possible objective functions, representing the user confidence about the function's properties such as amplitude and smoothness. The prior distribution is updated with each new measurement to produce a more accurate posterior distribution. The following point to be evaluated is determined by the acquisition function, which is based on the mean (μ) and standard deviation (σ) of the surrogate model. As an example, one can compute the maximum expected improvement (EI) (Močkus, 1975) over the current best result. The confidence intervals allows us to quantify model uncertainty and deciding when to stop the optimization search. The experiment design challenge is not unique to mRNA manufacturing, and IVT reactions in particular. Design problems are pervasive in both scientific and industrial settings. Bayesian optimization emerged has a powerful methodology for varied design problems (Shahriari et al., 2016) in domains like synthetic chemistry (Shields et al., 2021) or machine learning (Snoek et al., 2012), among many others.

In this article, we present for the first time a set of Machine Learning techniques to design, guide, and analyze experimental processes. In particular, our approach based on Bayesian optimization and explanation models is applied to the production of mRNA molecules in cell‐free IVT reaction. The data‐driven method we used allowed to achieve optimal mRNA production while dealing with a large parameter space, whilst significantly reducing the number of required experimental runs. Optimal IVT conditions were found in solely 60 runs with a maximum of 12 g . L^−1^ total mRNA being produced in approximately 2 h. The results obtained correspond to a IVT yield increase of a factor of two in half of the time, outperforming published industry standards and data reported in literature both in terms of achievable reaction yield and reduction of production time. These results reinforce the potential of Bayesian optimization to be applied on the optimization of (bio)chemical reactions for industrial applications.

## 2 METHODS

### 2.1 mRNA synthesis

#### 2.1.1 Template design

The mRNA template comprises the EGFP gene (GenBank Accession #AAB02572.1) flanked by 5′‐ and 3′‐UTR. The 5′‐UTR contains the T7 RNA polymerase promoter, an eukaryotic translation initiation factor 4 G eIF4G binding site and a Kozak consensus sequence (Tusup et al., 2019). Two β‐globin tandem repeats are used as a 3′‐UTR, followed by a 120 bp poly‐A, segmented with a 6 bp spacer (Trepotec et al., 2019). Two additional templates were constructed by fusing the Covid‐19 Receptor Bind Domain (RDB) gene (GenBank Accession #YP_009724390.1) and the Scocas9 (Huang et al., 2015) gene with the EGFP gene. All the templates are inserted in a puc57 vector with kanamycin resistance. Sequences used are found in Supporting Information: Table S2.

#### 2.1.2 Template production

DNA template is obtained by polymerase chain reaction (PCR). The reaction mixture contained 20 ng ml^−1^ of plasmid, 0.4 μM of forward and reverse primers, 0.2 mM dNTP mix; 1× Reaction buffer, 1× Stabilizer Solution, and 0.025 U μl^−1^ NZYProof DNA Polymerase (NZYTech). Primer sequences are found in Supporting Information: Table S3. The reaction is prepared to a final volume of 1000 μl and further split into single 20 μl reactions. The PCR reaction is initiated by a denaturation step at 95°C for 3 min, followed by 30 cycles of: (1) 30 s at 95°C; (2) annealing at 57.5°C for 30 s; (3) extension at 72°C for 60 s per kbp. The final extension is performed at 72°C for 60 s. The PCR product is purified and concentrated 20 times using Sera‐Mag Select (Cytiva) following the manufacturer instructions. Briefly, one volume of Sera‐Mag select is added to the pooled PCR reactions. After incubation, the supernatant is removed and the beads are washed two times with 85% v/v ethanol. The purified template is then eluted in water for injection (WFI), and quantified by UV spectroscopy using NanoDrop (Thermo Fisher Scientific).

#### 2.1.3 IVT reactions

IVT reactions are performed using T7 P&L RNA polymerase HC (Jena Biosciences). The purified PCR product is used as template, and natural NTPs (New England Biolabs) are used as substrate. *E. coli* inorganic pyrophosphatase (New England Biolabs) and RNase inhibitors (NZYTech), are added to the reaction volume. The reaction medium also contains magnesium acetate (heptahydrate) and magnesium chloride, spermidine (Alfa‐Aesar), dithiothreitol (Sigma), 40 mM Tris‐HCl (Fisher). The final volume of 20 μl was made up with WFI. Reactions were carried out in a thermocycler (Biometra). Reaction parameters were varied between set boundaries during optimization experiments (Table 1). The obtained samples were quantified using reverse‐phase high‐performance liquid chromatography (RP‐HPLC).

#### 2.1.4 mRNA purification

mRNA was purified using MEGAclear™ Transcription Clean‐Up Kit (Thermo Fisher Scientific) following manufacturing instructions with minor adjustments. Briefly, three 20 μl IVT reactions were pooled, 9 μl of TURBO™ DNase (Thermo Fisher Scientific), and 1 μl of 10× TURBO™ DNase Buffer (Thermo Fisher Scientific) were added, and the sample was incubated for 15 min at 37°C. 350 μl of binding buffer and 250 μl of absolute ethanol were added to the sample. The sample was loaded into the spin filter and centrifuged at 15,000 *g* for 1 min. The filter was washed and centrifuged in the previous conditions twice. For elution, 50 μl of elution buffer were added to the filter, incubated 5 min at 65°C, and centrifuged at 15,000 *g* for 1 min. Elution was performed twice. The 100 μl mRNA sample was further concentrated by precipitating with 10 μl of 5 M ammonium acetate and 270 μl absolute ethanol at −20°C for 30 min. The sample was centrifuged at 15,000 *g* for 10 min at 4°C. The obtained pellet was washed with 100 μl 75% ethanol and centrifuged using the previous conditions. The obtained pellet was let to dry and re‐suspended in 20 μl of elution buffer. The mRNA was quantified using Nanodrop 1 (Thermo Fisher Scientific) and the quality was evaluated by RP‐HPLC.

#### 2.1.5 IVT kinetics

IVT kinetics was studied with the reaction conditions described in Table 2 at volume of 65 μl. Reactions were carried out in a thermocycler (Biometra) and 5 μl samples were taken in a course of 5 h. The IVT reaction was stopped by dilute the sample 8 times in 1× pH 7 TAE buffer (100 mM Tris acetate, pH 7, 2.5 mM EDTA). The obtained samples were evaluated by RP‐HPLC and gel electrophoresis.

### 2.2 Analytical methods

#### 2.2.1 mRNA quantification

mRNA was quantified using RP‐HPLC using the method adapted from William Issa (2021). A 2.1 × 100 nm RP‐DNApac column and a guard column (3 × 10 nm) (Thermo Fisher Scientific) were used in a HPLC equipped with a column heater. Samples of 5 μl diluted eight times in 1× pH 7 TAE buffer (100 mM Tris acetate, pH 7, 2.5  mM EDTA), were injected in a pre‐equilibrated column with TAE buffer. The samples were eluted using 1× TAE with 25% (v/v) acetonitrile. The run was performed at 80°C, and the absorbance was monitored at 260 and 280 nm. The run conditions are present in Supporting Information: Table S4. The peak area corresponding to the elution of mRNA was considered for the evaluation. Calibration curves were constructed using purified mRNA samples with known concentrations in the range of 0.5–16 g · L^−1^.

Gel electrophoresis. Samples obtained from the IVT kinetic study were analyzed by gel electrophoresis. A 2% (w/v) agarose (Thermo Fisher Scientific) was prepared with 0.5× TBE buffer (Thermo Fisher Scientific) containing 5.5 mM of magnesium chloride (Thermo Fisher Scientific) and prestained with ethidium bromide (Thermo Fisher Scientific). The gel was loaded with a 1 μL mRNA sample diluted in 15 μL of WFI and 4 μL of 6× purple Loading Dye (New England Biolabs), and 4 μl of NZYDNA ladder III (NZYTech). The electrophoresis was performed at 100 V for 120 min using 0.5× TBE buffer, 5.5 mM MgCl_2_. The gels were scanned using an Axygen Gel Documentation System (Axygen).

### 2.3 Bayesian optimization

#### 2.3.1 Latin hypercube sampling (LHS)

LHS (McKay et al., 2000) is used to generate a series of initial experiments before the Bayesian optimization process is guided by the surrogate model. An implementation is available in the scikit‐optimize library (Head et al., 2020). LHS was used to sample an initial set of 16 reaction conditions to guarantee that the initial batch of reaction parameters do not overlap, and are sufficiently scattered over the candidate domains. The subsequent experiments are guided by the Bayesian optimization approach.

#### 2.3.2 Optimization cycle

**Figure 1:**
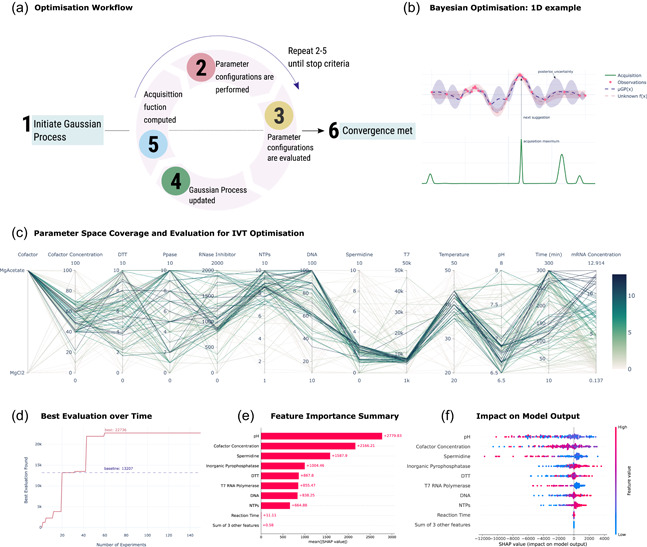
Bayesian optimization of messenger RNA (mRNA) in vitro transcription (IVT) reaction. (a) Bayesian optimization workflow. (b) One‐dimensional example of Bayesian optimization process using a Gaussian process surrogate model and corresponding acquisition function, maximized to select the next set of parameters to be tested. The surrogate model is plotted as the posterior mean, with the shaded region representing a posterior distribution uncertainty of 2σ units. (c) All parameter configurations for all the IVT experimental runs along with their respective evaluation in mRNA concentration (g · L^−1^). (d) Convergence plot depicting the best evaluation throughout all the IVT experimental runs, and convergence to the optimum. (e) Feature importance summary computed from the average SHapley Additive exPlanation (SHAP) values (Lundberg & Lee, 2017) computed for the Gaussian Process regressor predictions across all IVT experimental data. (f) Impact of feature value in model prediction value for the Gaussian Process regressor used as surrogate model in the Bayesian optimization process.

Two sequential experiments were performed. Overall, 16 reaction conditions were sampled and evaluated from an initial LHS design, and used to initialize the Bayesian optimization cycle (Figure 1a). After the surrogate model is initialized, three to five reaction conditions are suggested by the Bayesian model, the outcome for the IVT reactions for these conditions is evaluated and used to update the model each time. The optimization cycle was continued until no significant improvements in mRNA concentration were observed for a total of 150 reactions.

#### 2.3.3 Gaussian process (GP)

A GP was chosen to be the surrogate model for the Bayesian optimization process. A GP is a stochastic process such that any finite subcollection of random variables has a multivariate Gaussian distribution. A GP defines a prior distribution on functions f:X→R and can be thought of as the generalization of a Gaussian distribution over a finite vector space to a function space of finite dimension. Just as a Gaussian distribution is fully specified by its mean and covariance matrix, a GP is specified by a mean function m(x)=E[(f(x)] and a positive definite covariance (kernel) function k(x,x)=E[(f(x)−μ(x))(f(x)−μ(x)]. The chosen kernel greatly impacts the resulting distribution on functions and can correspond to strong assumptions about them (e.g., smoothness and differentiability). The squared exponential kernel is often the default choice for Gaussian process (GP) regression, but sample functions with this covariance function are unrealistically smooth for most practical optimization problems, as such, a Matérn 5/2 kernel Stein (1999) was chosen to be the kernel function for the GP:

(1) $$k_{M}^{5∕2}\left(x,x\prime \right)=\left(1+\frac{\sqrt{5}|x-x\prime |}{\ell }+\frac{5{\left(x-x\prime \right)}^{2}}{3\ell^{2}}\right)\text{exp}\left(-\frac{\sqrt{5}|x-x\prime |}{\ell }\right),$$

where ℓ is a length‐scale parameter. Matérn 5/2 yields twice differentiable sample functions, for example, like quasi‐Newton methods, without requiring the smoothness of the squared exponential. For the kernel hyperparameters (length scales, covariance amplitude, observation noise, and constant mean) a point estimate of these parameters was used by optimizing the marginal likelihood under the GPs. For a broader introduction to GPs (see Mackay, 1997; Rasmussen & Williams, 2005; Williams, 1997).

#### 2.3.4 Acquisition function

The acquisition function gives us the candidates for the next reaction conditions to be evaluated on each optimization cycle. The EI acquisition function (Močkus, 1975) is chosen for the Bayesian optimization process. The EI criterion is computed as follows: Let $f_{\max}=\max\left(y^{\left(1\right)},\text{…},y^{\left(n\right)}\right)$ be the current best function value. Let us model of uncertainty at y(x) as a normally distributed random variable Y with mean and standard deviation given by the surrogate model. Weighing all the improvements, the portion of the uncertainty density that extends beyond the current $f_{\max}$ by the corresponding density values, will give the EI. Formally, the improvement at a point x is given by the random variable $I=\min\left(0,f_{\max}+Y\right)$ and it models the uncertainty about the objective function value at x. The EI is the expected value:

(2) $$E\left[I\left(x\right)\right]\equiv E\left[\min\left(0,f_{\max}+Y\right)\right]=\left(f_{\max}+\hat{y}\right)\Phi \left(\frac{f_{\max}+\hat{y}}{s}\right)+\phi \left(\frac{f_{\max}+\hat{y}}{s}\right),$$

where $\hat{y}$ and s are the surrogate model prediction and its standard error at *x*, *Y* is Normal($\hat{y},s^{2}$), and ϕ(.) and Φ(.) are the standard normal density and distribution functions. Selecting values where x maximizes the EI acquisition function or from at random within a certain distance from the maximum improvement gives a balance between exploration and exploitation.

#### 2.3.5 SHAP (SHapley Additive exPlanations) of GP estimator

SHAP (Lundberg & Lee, 2017) is a method based on the game theory concept of optimal Shapley values that views any explanations of a model's prediction as a model itself, an explanation model. This method was used to evaluate the predictions of GP estimator. Independent variable values are interpreted as players in a coalition game from which Shapley values are computed. This approach unifies other methods as additive feature attribution methods, where an explanation model is a linear function of binary variables:

(3) $$g\left(z\prime \right)=\phi_{0}+\sum_{j=1}^{M}\phi_{j}z_{j\prime }^{\prime }$$

where $z\prime \in {\left\{0,1\right\}}^{M}$, M is the number of simplified input features, and $\phi_{i}\in R$. The explanation model attributes an effect $\phi_{i}$ to each feature, and summing the effects of all feature attributions approximates the output f(x) of the original model.

The exact computation of SHAP values is challenging (Lundberg & Lee, 2017). To generate explanations for the GP predictions, approximations are computed using a model‐agnostic permutation‐based explanation model that uses the Shapley sampling values method (Štrumbelj & Kononenko, 2013).

#### 2.3.6 Surrogate model comparison

The generalization error of Random Forests (RF) (Breiman, 2004) and Gradient Boosting Machines (GBM) (Friedman, 2001) was compared to the generalization error for the Gaussian Process used throughout the experimental runs. Leave‐one‐out cross validation was performed on the reaction data (150 configurations including replicated runs). The surrogate model is trained on each data fold of size *n*− 1 and the absolute prediction error is measured on the remaining data point. Supporting Information: Figure S1 shows the distribution of errors for GP, RF, and GBM.

## 3 RESULTS

### 3.1 IVT optimization workflow

Briefly, in an IVT reaction, a RNA polymerase uses a target DNA template to synthesize the complementary RNA molecule using NTPs as substrate. In this study, 12 reaction parameters were identified that could potentially influence the reaction outcome: enzyme activity (T7 RNA polymerase and inorganic phosphatase); the concentration of RNase inhibitor, DNA template, NTPs, spermidine and dithiothreitol (DTT); the type of cofactor (e.g., magnesium acetate vs magnesium chloride) and their respective concentration; reaction pH, temperature and reaction time (Table 1). The reaction and process parameters are summarized in Table 1. The mRNA concentration obtained in each experimental run suggested by the model is fed into the Bayesian optimizer with the initial reaction conditions randomized. In each imposed optimization cycle, the model is updated and new experimental conditions are suggested based on the model knowledge of the process, that is, the maximum mRNA to be produced (Figure 1a). Specifically, the optimization loop proceeds as follows:

1. A GP surrogate model is initialized with a covariance function, specifically, Matérn 5/2 kernel.
2. The surrogate model is fed with a batch of random initial reaction conditions taken from a latin hypercube sampling (LHS) design (McKay et al., 2000).
3. The selected reaction conditions are experimentally performed and the produced mRNA quantified.
4. The surrogate model is updated with the mRNA produced in each of the reaction conditions evaluated to construct a posterior distribution.
5. The EI acquisition function (Močkus, 1975) is computed based on the surrogate model and its maximum value is used to suggest the proceeding reaction configurations (Figure 1b).
5. Steps 3 and 4 are repeated until the selected convergence criteria is met, this can be the maximum budget for the total number of experiments is reached *(maximum budget*), and/or a number of experimental runs are performed without statistically significant improvement.

**Table 1:** In vitro transcription (IVT) reaction parameters and evaluation metric (see also Figure 1c)

Name	Units	Type	Domain/range
Cofactor	Cofactor choice	Categorical	MgAcetate, MgCl_2_
Cofactor concentration	mM	Real number	[0, 100]
DTT	mM	Real number	[0,10]
RNase inhibitor	U ml^−1^	Integer	[0,2000]
NTPs	mM	Real number	[1.0,10.0]
DNA template	nm	Integer	[10, 100]
Inorganic pyrophosphatase	U ml^−1^	Integer	[0, 10]
Spermidine	mM	Real number	[0, 10]
T7 RNA polymerase	U ml^−1^	Integer	[1000, 50,000]
Temperature	°C	Integer	[20,50]
Reaction time	min	Integer	[10, 300]
pH	—	Real number	[6.5,8]
Evaluation	g_mRNA_ L−1	Integer	⋯

Abbreviations: DTT, dithiothreitol; mRNA, messenger RNA; NTP, nucleoside triphosphate.

### 3.2 Optimization analysis

The progress of the optimization procedure can be followed in multiple ways. The exploration of the reaction parameter space can be performed with the aid of a parallel coordinate plot (Figure 1c). This plot provides an overview over the possible optimal reaction parameter ranges across the entire optimization process. The best reaction conditions are also evaluated over time, being used to determine if significant progress is being made in the optimization cycle. By analyzing the parallel coordinate plot for the entire optimization experiment, it is possible to observe that the optimal mRNA production is obtained when the concentration of magnesium acetate concentration is between 40 and 70 mM, the concentration of NTPs is above 7 mM, spermidine concentration is between 1 and 3 mM, the volumetric activity of the T7 RNA polymerase (T7 RNAP) is between 6000 and 8000 U. ml^−1^, the reaction temperature is between 37 and 45°C, and the initial pH is lower than 7.5. Reactions conditions that produced a maximum amount of mRNA were found in the first 60 runs of the optimization routine (Figure 1d). Several reaction conditions were obtained that yielded more than 10 g_mRNA_ · L^−1^.

To better understand the IVT reaction optimization, two different mechanisms were used. First, the model predictions in terms of expected mRNA IVT production and uncertainty boundaries with the reaction empirical evaluations were compared. Second, explanation models were built for the GP surrogate model (Figure 1e,f). The SHAP (Lundberg & Lee, 2017) gives the overall parameter importance regarding parameter impact in model predictions. As the surrogate model is fed with increasing data, and it converges to the unknown function (i.e., the IVT reaction being modeled), the explanations become more informative about the real impact of different reaction parameters on the amount of mRNA being produced. The evaluation of the coordinate plot combined with these mechanisms allowed to infer the impact of particular parameter on surrogate model predictions. The pH has a high impact (positive) on the reaction outcome, in particular between 6.5 and 7.5. This is followed by the cofactor concentration with optimal range set between 40 and 60 mM. High inorganic pyrophosphatase and DTT concentrations impact positively the reaction. This is also observed for higher concentrations of DNA and NTPS, above 40 nM and 7 mM, respectively. T7 RNAP presence impacts the model, but at lower enzyme volumetric activities, between 6000 and 8000 U · mL^−1^, which will produce ultimately more mRNA. Surprisingly, high spermidine concentration impact negatively the model, and optimum values used should be in the range of 1 and 3 mM (Figure 1c,e,f).

### 3.3 IVT kinetics

In the experiments performed, the reaction time was not explicitly optimized. Nevertheless, the six best reactions conditions that produced mRNA exceeding 10.7 g · L ^−1^ (Table 2) were evaluated in term of reaction profile. These reactions were compared with a benchmark reaction conditions, corresponding to the reaction parameters listed in the Moderna patent (Bancel et al., 2016), which result in an expected mRNA production yield of 5 g ·  L^−1^.

**Table 2:** mRNA production reaction parameters and mRNA concentration for highest production conditions after Bayesian optimization (Reactions 1–6) compared to the benchmark reaction (Bancel et al., 2016) condition (Reaction 7)

Reaction	1	2	3	4	5	6	7
Cofactor C (mm)	60.00	48.46	41.79	59.87	49.28	40.00	40.00
DTT (mm)	7.09	3.85	5.57	9.85	5.27	3.99	5.00
RNase I (U mL^−1^)	829	1072	1217	986	1474	1045	1000
NTPs (mm)	8.57	8.81	9.89	8.50	7.75	9.29	7.50
DNA template (nm)	61	100	89	100	89	72	40
Ppase (U mL^−1^)	10	9	5	2	8	7	1
Spermidine (mm)	2.65	1.35	2.24	1.31	2.25	2.03	1.00
T7 RNAP (U mL^−1^)	7346	7320	6607	6166	7743	7748	7000
Temperature (°C)	43	39	44	40	44	44	37
Time (min)	263	98	120	148	121	279	240
pH	6.89	6.80	6.65	6.78	6.67	6.60	8.00
mRNA C (g · L^−1^)	12.61	10.76	11.76	12.27	12.18	11.52	7.64
	±0.82	±0.47	±0.66	±0.77	±0.98	±0.23	±0.87

Abbreviations: DTT, dithiothreitol; mRNA, messenger RNA; NTP, nucleoside triphosphate.

**Figure 2:**
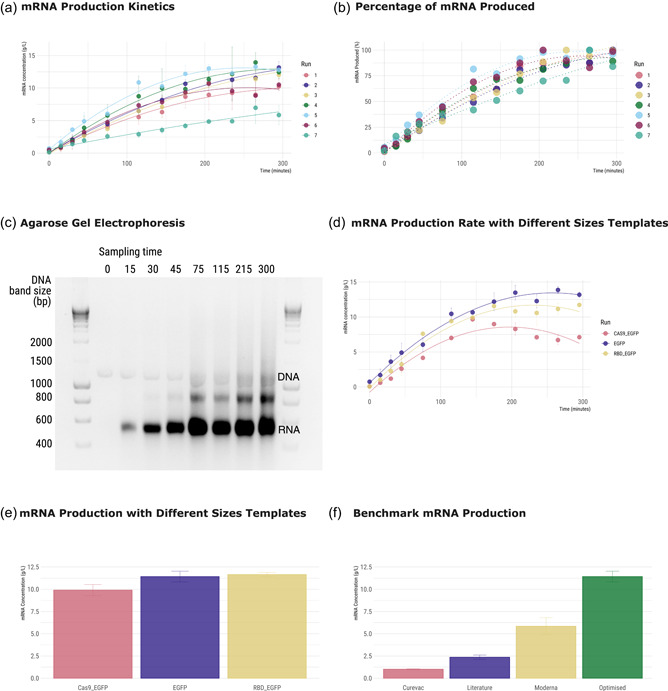
Messenger RNA (mRNA) production analysis. (a) mRNA production profile of the six best runs obtained by Bayesian optimization and the benchmark reaction 19 for a time course of 5 h. Error bars represent standard deviation obtained for each point. A second order polynomial function was used as a trendline for visualization purposes. (b) Percentage of mRNA produced as a function of minutes of reaction time considering 100% the highest mRNA concentration produced for each set of runs. Error bars represent standard deviation obtained for each point. A second order polynomial function was used as a trendline for visualization purposes. (c) Agarose gel electrophoresis analysis of the reaction mixture at the defined setpoints for the best production run (Run 5). (d) mRNA production profile for a time course of 5 h using the best run parameters (Run 5), and templates with the different sizes (EGFP—1195 bp; RBD_EGFP—1864 bp) Cas9_EGFP—5299 bp). Error bars represent standard deviation obtained for each point. A second order polynomial function was used as a trendline for visualization purposes. (e) mRNA production using the parameters of the Run 5 and 2 h of reaction time with the three different size templates (EGFP, RBD_EGFP, Cas9_EGFP). (f) mRNA production concentration ($g_{\text{mRNA}}\;\cdot \;L^{-1}$) using EGFP template for the following runs: optimized—Run 5 and 2 h of reaction time; Moderna, Inc. (Bancel et al., 2016) and Curevac N.V. (Wochner et al., 2021) patent conditions and literature conditions (Henderson et al., 2021).

To obtain the reaction profile, samples were taken over the course of 5 h (Figure 2a). All the reactions found by the optimization process outperformed the chosen baseline reaction, producing at least 10 $g_{\;\text{mRNA}}\;\cdot \;L^{-1}$ of mRNA. Although during the optimization runs, reactions 1 and 4 produced the highest amount of mRNA, ultimately reaction 5 achieved the highest yield in less time as after 2 h (Supporting Information: Table S1), corresponding to a final concentration of $10.65\pm 0.01\;g_{\;\text{mRNA}}\;\cdot \;L^{-1}$, that is, 80% of the maximum mRNA produced (Figure 2b). The quality of mRNA produced was also assessed by agarose gel electrophoresis (Figure 2c) and HPLC analysis (Supporting Information: Figure S3). After approx. 115 min no significant changes in mRNA amount are detected, confirming that reaction performance peaks at the 2 h mark. A second band is observed to increase after 115 min that may correspond to reaction by‐products such as double‐strand mRNA (dsmRNA) or aberrant mRNA. HPLC analysis shows that at reaction completion the ratio $\mathrm{dsRNA}/{\mathrm{RNA}}_{\mathrm{total}}$ is $0.05\pm 0.003\;\mathrm{mg}\;\cdot \;{\mathrm{mg}}^{-1}$.

### 3.4 IVT performance validation

To validate the optimized reaction performance, we compared the mRNA production using different size templates. The templates containing  *EGFP* gene, the Covid‐19 Receptor Bind Domain fused to *EGFP* (RDB_EGFP), and the *Cas9* gene also fused to *EGFP* (Cas9_EGFP), with 1195, 1864, and 5299 bp, respectively. The differences between reaction profile (Figure 2d), and concentration of total mRNA produced after 2 h (Figure 2e) were investigated. Changing the size of the template does not have an impact on the reaction outcome using the optimized conditions. All the evaluated templates sizes produced over 10 g · L^−1^ of mRNA. However, with larger templates the mRNA concentration tends to reduce from a concentration of $9.7\pm 0.29\;g\;\;L^{-1}$ (145 min of reaction time) to a concentration of $7.1\pm 0.19\;g\;\cdot \;L^{-1}$ (end of reaction). In spite of this reduction, $10\;g_{\;\text{mRNA}}\;.\;L^{-1}$ is still produced within 2 h of reaction time.

### 3.5 Optimal IVT production and comparison

The highest mRNA producing reaction condition was compared with different reaction conditions reported in the literature and patents (Bancel et al., 2016; Henderson et al., 2021; Wochner et al., 2021) (Figure 2f). Within 2 h of reaction time, a total amount of $12\;g_{\;\text{mRNA}}\;\cdot \;L^{-1}$ was obtained, outperforming the benchmark reaction by twofolds (Table 2).

## 4 DISCUSSION

IVT reaction optimization has become increasingly important not only due to the growing interest in using RNA molecules in a number of diagnostic and therapeutic applications, but especially due to the rise of mRNA vaccine technology in recent years. However, existing optimization experiments are focused on reaction modelling (Breckenridge & Davis, 2000; Kern & Davis, 1997, 1999; Young et al., 1997); or on DoE (Samnuan et al., 2021) methodologies. Optimization methods only consider the exploration of small parameter spaces and often assume that the relationships between reaction parameters are given by pre‐established enzymatic dynamic models. In this study, we demonstrate the effectiveness of the Bayesian optimization methodology when applied to the production of mRNA by IVT, a multicomponent reaction that depends on 12 different reaction parameters. Using this method, the maximum amount of mRNA produced was found in only 60 experimental runs. Interpretability techniques, in particular, explanation models based on Shapley Values (Lundberg & Lee, 2017) for the Gaussian Process surrogate model were also used. The combination of the obtained results with the explanation models allowed to bridge these explanations and/or interpretations with pre‐existing knowledge about the IVT reaction being modeled optimized.

For any optimization problem, the most important metric to be defined is the objective evaluation. In the case of the IVT reaction, the goal is to maximize the quantity of mRNA produced for a specific set of reaction parameters. To achieve this, mRNA concentration was used as the surrogate metric. The initial set of 16 reactions to be evaluated were sampled randomly using. Latin Hypercube Sampling (LHS) (McKay et al., 2000). This is a type of stratified Monte Carlo (MC) sampling where the range of each variable is partitioned into *N* nonoverlapping intervals on the basis of equal probability size 1/N. The number of partitions is equal to the number of required samples. LHS guarantees that the initial parameter configurations do not overlap and are sufficiently scattered over the target parameter space. This is often important to guarantee that the model is seeded with different regions of the target response surface. Nevertheless, since sampling overlapping configurations is extremely unlikely with 12 parameters and there is a small number of initial experiments, this sampling strategy is as good as random uniform.

Public data sets on IVT reactions for mRNA production are currently non‐existent, making the estimation of the impact of the different components that compose our Bayesian optimization approach difficult. Therefore, a practical starting point is chosen for the surrogate model, acquisition function, and hyperparameters, namely: a GP surrogate model with a Matérn 5/2 kernel and the EI acquisition function. These settings do not make assumptions about the underlying structure of the target problem while still being capable of tackling a wide range of domains. Nevertheless, there are multiple sensible choices for surrogate models besides a GP. A post‐hoc comparison of generalization error between RF (Breiman, 2004), Gradient Boosting Machines (GBM) (Friedman, 2001), and GP (Mackay, 1997) was performed and found no significant differences between them in terms of estimated generalization power (Supporting Information: Figure S1). Techniques such as deep kernel learning (Wilson et al., 2016) can be used to combine the structural properties of deep learning architectures with the non‐parametric flexibility of Gaussian Processes, allowing the creation of specialized kernels for this (or similar) domains.

Finally, in terms of the optimization process, no reaction conditions were found after 60 runs that could increase the total mRNA concentration (Figure 1d). The proposed optimization only considered the maximization of mRNA production as a goal. Multiple‐objective optimization can be used in the future to obtain the Pareto frontier of IVT reactions using multiple metrics. This would allow to find sets of optimal trade‐offs, which can include minimizing reagent concentrations, reaction time, reaction cost and dsRNA production, while maximizing reaction yield.

By combining the interactive nature of the optimization cycle and the insights gained from the explanation models, it was possible to both validate the model predictions and and study the IVT reaction itself. This allowed the construction of a more coherent view of the dynamics of IVT reactions in general. IVT reactions employ a RNA polymerase and a DNA template to generate RNA using the NTPs as substrate and magnesium as a cofactor. T7 RNA polymerase (T7 RNAP) is a 98 kDa and single subunit enzyme that catalyzes RNA synthesis of very long transcripts without auxiliary transcription factors (Borkotoky et al., 2017; Borkotoky & Murali, 2018). Due to its characteristics, T7 RNAP is widely used for the production of mRNA by IVT. Parameters importance and their impact in the surrogate model predictions combined with the information given by the coordinate plot were compared with existing literature (Figure 1c,e,f). The model predicted that pH, cofactor concentration, spermidine concentration, and inorganic pyrophosphatase have the most impact on the reaction outcome. These are followed by DTT concentration, T7 RNAP activity, DNA concentration, NTPs concentration, and reaction time (Figure 1e). The least impactful parameters predicted include the reaction temperature, the type of co‐factor and the RNase inhibitor concentration. The model predicts that a low pH value significantly and positively impacts the mRNA production (Figure 1e,f). Typically, IVT reactions are performed using higher pH values, for example, 7.9 or 8 (Bancel et al., 2016; Henderson et al., 2021). However, by exploring a wider range of pH, it was found that values between 6.5 and 7.2 improve the transcription rate. These results are in line with previous studies where an optimal transcription rate is reported for values of pH between 7.0 and 7.5 (Kern & Davis, 1997).

Magnesium also plays an important role in the IVT reaction since it is required to bind T7 RNAP enzyme to the DNA template (Gunderson et al., 1987). Additionally, magnesium‐NTP complexes are used to form phosphodiester bonds with the RNA chain (Breckenridge & Davis, 2000) where a pyrophosphate is released as a by‐product. Depending on the concentration of Mg^2+^ present in the reaction, free pyrophosphate can cross‐link with free Mg^2+^ and precipitate due to the formation of long aggregates. The results showed that the mRNA production rate is increased with concentrations of magnesium acetate between 40 and 60 mM. It is important to maintain a high concentration of free Mg^2+^ to ensure that the cofactor does not limit the reaction. An optimal range between 50 and 60 mM was predicted in Breckenridge and Davis (2000). Additionally, the presence of counter ions can also inhibit mRNA production. Through the optimization process it was also found that magnesium acetate is preferred since it can be used in higher concentrations than magnesium chloride. These findings are in line with previous reported results (Kern & Davis, 1997; Samnuan et al., 2021).

The pyrophosphate by‐product may inhibit IVT as it reduces the free Mg^2+^. To avoid this, an inorganic pyrophosphatase (PPase) can be used to catalyze the hydrolysis of pyrophosphate. This leads to the formation of orthophosphate, releases Mg^2+^, and, ultimately, increases mRNA production (Kern & Davis, 1997). The use of PPase in IVT is not consensual as this enzyme potentially does not impact mRNA production if the Mg^2+^ is present in sufficient concentration (Kern & Davis, 1997, 1999). However, our findings show that higher concentrations of PPase positively influence the mRNA production (Figure 1f). This has been previously observed when PPase was used in volumetric activities between 5 and 10 U mL^−1^ (Frugier et al., 1994; Gosule & Schellman, 1978). This positive influence can be particularly observed when using high concentration of NTPs (Kern & Davis, 1999). We hypothesize that PPase is important to maintain a threshold concentration of Mg^2+^ and hence sustain a high mRNA production rate.

Spermidine is an aliphatic polyamine with high affinity toward nucleic acids that neutralizes negative charges, and consequently, promotes condensation and aggregation of DNA (Gosule & Schellman, 1978). In the IVT reaction, spermidine plays an important role in the transcription initiation as it stabilizes the DNA‐enzyme complex (Frugier et al., 1994). Its presence in IVT reaction is fundamental as it can lead to an increase of up to 10 times when using T7 RNAP as enzyme (Watanabe et al., 1983). Here we found that spermidine can also have an inhibitory effect when present in high concentrations. However, when used in concentrations between 1 and 3 mM, it influences the mRNA production positively. These results are in line with optimal conditions found in reported data (Fuchs, 1976; Moussatché, 1985).

As previously described, there is a close relation between the substrate concentration and the cofactor, as NTPs and Mg^2+^ form complexes that are added to the nascent mRNA chains. NTPs concentration must be high enough to promote the reaction. However, the concentration increase beyond a certain value does not have a significant influence on the reaction output (Breckenridge & Davis, 2000). Our findings suggest that NTPs concentration above 7 mM have a positive impact on mRNA production, and are comparable with literature (Pokrovskaya & Gurevich, 1994).

We observed that the optimal T7 RNAP volumetric activity is between 6000 and 8000 U · mL^−1^, and that activities above this point have a negative impact on the reaction yield. This shows that increasing enzyme volumetric activity does not translate into higher mRNA production due to possible diffusion limitation and solubility challenges. Another important parameter is the template concentration. It should be high enough to guarantee that it is not the limiting factor in the reaction itself. We observed that DNA concentrations above 40 nm should be used to guarantee optimal production. DTT is a reducing agent that plays an important role to maintain the enzymes activities during the transcription. Reaction temperature, although it does not seem to have an impact on the reaction, affects the binding of the enzyme to the template promoter. To achieve total binding (Oakley et al., 1975), temperatures of at least 37°C should be used. Additionally, high temperature could also reduce the formation of ds‐mRNA (Wu et al., 2020), a reaction by‐product that leads to a decrease in vaccine efficiency when present in the final product (Mishra et al., 2022). The model predicts that the optimal reaction temperature in the system should be between 37°C and 44°C.

A larger and more coherent picture of the impact of reaction parameters on IVT has emerged from the optimization results herein presented. Multiple reaction conditions were found that lead to mRNA concentrations higher than 10 g · L^−1^. In terms of production rate, we observed that the reaction time can be reduced to 2 h. Ultimately, we found a set of IVT reaction parameters able to produce 12 g_mRNA_ · L^−1^ (Figure 2f). This means a twofold increase in half of time when compared with the reported literature. Additionally, increasing template size did not have an impact on production using the optimized conditions as all the templates evaluated produced more than 10 g_mRNA_ · L^−1^ of mRNA (Figure 2e). Nevertheless, increasing the reaction time above two hours for longer DNA templates is not beneficial as a decrease in mRNA concentration is observed (Figure 2d). This can be explained by the formation of a precipitate which could precipitate the mRNA already formed, and consequently, to the generation of aberrant mRNA species.

Another important point is the quality of the mRNA produced. This metric was not considered in the optimization evaluation because it introduces a new criterion, transforming the problem into a multi‐objective optimization scenario. While this is the natural progression of the present work, a single objective setting allowed us to validate all the components involved before considering trade‐off decisions when searching for the optimal reaction conditions. The presence of by‐products in the final product has a strong impact both in mRNA translation efficiency within the patient cells and in the immunostimulatory profile (Rosa et al., 2021). We have observed that the dsmRNA is produced alongside the ssmRNA after a threshold concentration is reached (Supporting Information: Figure S3). At reaction completion, a maximum ratio of dsmRNA to total mRNA ($\mathrm{dsRNA}/{\mathrm{RNA}}_{\mathrm{total}}$) obtained is $0.05\pm 0.003\;\mathrm{mg}\;\cdot \;{\mathrm{mg}}^{-1}$. Similar results have been reported (Nelson et al., 2020). The mRNA quality attributes can be further studies by a number of analytical techniques such as electrophoresis, HPLC or ELISA (Poveda et al., 2019). Additionally, mRNA structure and identity could be also analyzed using circular dichroism (CD), RT‐PCR or next‐generation sequencing (World Health Organization, 2020). Nonetheless, this was out of scope for the present study.

## 5 CONCLUSION

Overall, by using Bayesian optimization, we were able to increase the mRNA IVT production twofold, up to 12 g_mRNA_ · L^−1^, in under two hours when compared to published industry standards and data reported in literature. This optimization approach proved to be cost‐effective, as it only required 60 reactions to achieve optimal parameter combinations. Using Machine Learning techniques in combination with the experimental observations and intuitions, were the key to ultimately detect human error in the reaction preparation and assess model improvement over time. This reveals the importance of interactivity and explainability in a scientist‐in‐the‐middle approach to problems being solved with techniques from Machine Learning. This allowed to better understand the IVT reaction parameter's impact on the model increasing the existing know‐how on IVT reactions to create in the future more efficient and flexible processes. The results obtained can potentially increase the global manufacturing capacity of mRNA vaccines. Additionally, these results reinforce the potential of Bayesian optimization to be applied on the optimization of (bio)chemical reactions for industrial applications.

## AUTHOR CONTRIBUTIONS

Sara Sousa Rosa and Davide Nunes were responsible for the conceptualization of the project and formulation of the overarching research goals, the development of the methodology for the application of Bayesian optimization to IVT reactions for the production of mRNA, formal analysis of the result data, the preparation and visualization of the result data, writing the initial draft, and reviewing and editing the final version of the paper. Sara Sousa Rosa was responsible for implementing and performing the IVT reaction experiments, mRNA quantification using RP‐HPLC, experiments related to the IVT production rate, and validation of benchmark reactions. Sara Sousa Rosa and Marco P. C. Marques were responsible for the analysis of the optimization and kinetics setup. Davide Nunes was responsible for the data curation, implementation of the optimization software platform including the Bayesian optimization components, visualization, and explanation techniques, and post‐hoc validation of the models used. Luis Antunes, Ana M. Azevedo, Duarte M. F. Prazeres, and Marco P. C. Marques were responsible for editing, reviewing, and offering critical commentary on the paper. Ana M. Azevedo, Duarte M. F. Prazeres, and Marco P. C. Marques were responsible for supervising the work of Sara Sousa Rosa.

## CONFLICT OF INTEREST

The authors declare no conflict of interest.

## Supporting information

Supplementary Information

## Data Availability

The data along with the software for its analysis supporting the findings of this study are available from Nunes and Rosa (2022).
