# Supplementary material for: Maximizing mRNA vaccine production with Bayesian optimization
Source: Biotechnol Bioeng. 2022 Sep 5:10.1002/bit.28216. Online ahead of print. doi: 10.1002/bit.28216 (PMC9539360; doi:10.1002/bit.28216)
Supplement: Supplementary file 1 — Supplementary Information [file BIT-9999-0-s001.pdf]

1 Supplementary information - Rosa et al., Maximizing mRNA Vaccine Production with Bayesian Optimization

Supplementary Table S1: Optimization of mRNA production in concentration ($g_{mRNA}.L^{-1}$) for each run (1-7) presented in Table 2 and the values obtained in the kinetics studies (Figure 2a) at 80% of maximum production obtained during the kinetics reactions for each run and the corresponding times (min).

	Optimization Studies		Kinetics Studies	
Run	Concentration $g.L^{-1}$	Time (min)	Concentration $g.L^{-1}$	Time (min)
1	12.61 ± 0.82	263	8.19 ± 1.76	175
2	10.76 ± 0.47	98	10.63 ± 0.6	175
3	11.76 ± 0.66	120	9.27 ± 0.51	175
4	12.27 ± 0.77	148	11.34 ± 1.19	205
5	12.18 ± 0.98	121	10.85 ± 0.26	115
6	11.52 ± 0.23	279	8.52 ± 0.93	145
7	7.64 ± 0.87	240	5.57 ± 0.3	235

Supplementary Table S2: Sequences in the 5'UTR, 3'UTR Poly-A used and the gene of interest used in the three DNA templates EGFP, RDB_EGFP, Cas9_EGFP.

Name	Size (bp)	Sequence
5'UTR	65	TAA TAC GAC TCA CTA TAG GGA CTC ACT ATT TGT TTT CGC GCC CAG TTG CAA AAA GTG TCG CCA CC
3'UTR	284	GAG AGC TCG CTT TCT TGC TGT CCA ATT TCT ATT AAA GGT TCC TT TGT TCC CTA AGT CCA ACT ACT AAA CTG GGG GAT ATT ATG AAG GGC CTT GAG CAT CTG GAT TCT GCC TAA TAA AAA ACA TTT ATT TTC ATT GCT GCG TCG AGA GCT CGC TTT CTT GCT GTC CAA TTT CTA TTA AAG GTT CCT TTG TTC CCT AAG TCC AAC TAC TAA ACT GGG GGA TAT TAT GAA GGG CCT TGA GCA TCT GGA TTC TGC CTA ATA AAA AAC ATT TAT TTT CAT TGC TGC GTC
Poly-A	126	AAA AAA AAA AAA AAA AAA AAA AAA AAA AAA AAA AAA AAA AAA AAA AAA AAA AAA AAA AAA ATG CAT AAA AAA AAA AAA AAA AAA AAA AAA AAA AAA AAA AAA AAA AAA AAA AAA AAA AAA AAA AAA
**Genes**		
EGFP	720	ATG GTG AGC AAG GGC GAG GAG CTG TTC ACC GGG GTG GTG CCC ATC CTG GTC GAG CTG GAC GGC GAC GTA AAC GGC CAC AAG TTC AGC GTG TCC GGC GAG GGC GAG GGC GAT GCC ACC TAC GGC AAG CTG ACC CTG AAG TTC ATC TGC ACC ACC GGC AAG CTG CCC GTG CCC TGG CCC ACC CTC GTG ACC ACC CTG ACC TAC GGC GTG CAG TGC TTC AGC CGC TAC CCC GAC CAC ATG AAG CAG CAC GAC TTC TTC AAG TCC GCC ATG CCC GAA GGC TAC GTC CAG GAG CGC ACC ATC TTC TTC AAG GAC GAC GGC AAC TAC AAG ACC CGC GCC GAG GTG AAG TTC GAG GGC GAC ACC CTG GTG AAC CGC ATC GAG CTG AAG GGC ATC GAC TTC AAG GAG GAC GGC AAC ATC CTG GGG CAC AAG CTG GAG TAC AAC TAC AAC AGC CAC AAC GTC TAT ATC ATG GCC GAC AAG CAG AAG AAC GGC ATC AAG GTG AAC TTC AAG ATC CGC CAC AAC ATC GAG GAC GGC AGC GTG CAG CTC GCC GAC CAC TAC CAG CAG AAC ACC CCC ATC GGC GAC GGC CCC GTG CTG CTG CCC GAC AAC CAC TAC CTG AGC ACC CAG TCC GCC CTG AGC AAA GAC CCC AAC GAG AAG CGC GAT CAC ATG GTC CTG CTG GAG TTC GTG ACC GCC GCC GGG ATC ACT CTC GGC ATG GAC GAG CTG TAC AAG TAA
RDB_EGFP	1389	ATG CGT GTG CAG CCG ACT GAA TCC ATC GTG CGC TTC CCG AAC ATC ACG AAC CTG TGC CCG TTC GGT GAA GTG TTC AAC GCA ACC CGT TTC GCA TCG GTT TAT GCA TGG AAC CGT AAA CGC ATC AGC AAT TGC GTG GCG GAT TAC TCC GTT CTT TAT AAT AGC GCA TCG TTT AGC ACC TTC AAA TGC TAT GGT GTG AGT CCA ACC AAA CTT AAT GAT CTG TGC TTC ACG AAT GTG TAC GCA GAC AGT TTC GTC ATC CGT GGC GAC GAG GTG CGC CAG ATT GCC CCG GGC CAA ACG GGC AAG ATC GCA GAT TAC AAT TAT AAA TTA CCG GAT GAC TTC ACG GGT TGT GTT ATT GCA TGG AAC AGC AAC AAT CTG GAT AGC AAA GTT GGT GGT AAC TAC AAT TAT CTG TAT CGC CTG TTC CGC AAA AGC AAC TTG AAA CCG TTT GAA CGT GAC ATC AGC ACG GAA ATT TAT CAG GCC GGT TCC ACG CCG TGT AAT GGT GTG GAA GGT TTC AAT TGC TAC TTC CCA CTT CAG AGT TAT GGC TTT CAG CCG ACG AAC GGT GTC GGT TAC CAG CCG TAT CGT GTG GTT GTC CTG AGC TTC GAA CTG TTG CAC GCG CCG GCA ACG GTG TGT GGC CCG AAA AAA TCC ACG AAT CTG GTG AAG AAC AAA TGC GTG AAC TTT GTG AGC AAG GGC GAG GAG CTG TTC ACC GGG GTG GTG CCC ATC CTG GTC GAG CTG GAC GGC GAC GTA AAC GGC CAC AAG TTC AGC GTG TCC GGC GAG GGC GAG GGC GAT GCC ACC TAC GGC AAG CTG ACC CTG AAG TTC ATC TGC ACC ACC GGC AAG CTG CCC GTG CCC TGG CCC ACC CTC GTG ACC ACC CTG ACC TAC GGC GTG CAG TGC TTC AGC CGC TAC CCC GAC CAC ATG AAG CAG CAC GAC TTC TTC AAG TCC GCC ATG CCC GAA GGC TAC GTC CAG GAG CGC ACC ATC TTC TTC AAG GAC GAC GGC AAC TAC AAG ACC CGC GCC GAG GTG AAG TTC GAG GGC GAC ACC CTG GTG AAC CGC ATC GAG CTG AAG GGC ATC GAC TTC AAG GAG GAC GGC AAC ATC CTG GGG CAC AAG CTG GAG TAC AAC TAC AAC AGC CAC AAC GTC TAT ATC ATG GCC GAC AAG CAG AAG AAC GGC ATC AAG GTG AAC TTC AAG ATC CGC CAC AAC ATC GAG GAC GGC AGC GTG CAG CTC GCC GAC CAC TAC CAG CAG AAC ACC CCC ATC GGC GAC GGC CCC GTG CTG CTG CCC GAC AAC CAC TAC CTG AGC ACC CAG TCC GCC CTG AGC AAA GAC CCC AAC GAG AAG CGC GAT CAC ATG GTC CTG CTG GAG TTC GTG ACC GCC GCC GGG ATC ACT CTC GGC ATG GAC GAG CTG TAC AAG TAA

Cas9_EGFP	4824	ATG GAC AAG AAG TAC TCC ATC GGC CTG GAC ATC GGC ACC AAC TCC GTC GGC TGG GCC GTC<br>ATC ACG GAC GAG TAC AAG GTC CCG TCG AAG AAG TTC AAG GTG CTG GGC AAC ACG GAC CGC<br>CAC AGC ATC AAG AAG AAC CTC ATC GGC GCC CTC CTG TTC GAC AGC GGC GAG ACG GCC GAG<br>GTG ACC CGC CTG AAG CGC ACG GCG CGG CGG CGG TAC ACG CGC CGC AAG AAC CGC ATC TGC<br>TAC CTC CAG GAG ATC TTC TCC AAC GAG ATG GCG AAG GTC GAC GAC TCG TTC TTC CAC CGG<br>CTG GAG GAG TCC TTC CTG GTC GAG GAG GAC AAG AAG CAC GAG CGG CAC CCC ATC TTC GGC<br>AAC ATC GTG GAC GAG GTG GCC TAC CAC GAG AAG TAC CCG ACC ATC TAC CAC CTC CGG AAG<br>AAG CTG GTG GAC TCG ACC GAC AAG GCC GAC CTG CGC CTG ATC TAC CTG GCC CTC GCC CAC<br>ATG ATC AAG TTC CGC GGC CAC TTC CTC ATC GAG GGC GAC CTG AAC CCG GAC AAC TCG GAC<br>GTC GAC AAG CTG TTC ATC CAG CTC GTG CAG ACC TAC AAC CAG CTC TTC GAG GAG AAC CCG<br>ATC AAC GCC TCC GGC GTG GAC GCG AAG GCC ATC CTG TCG GCC CGC CTC TCG AAG TCC CGG<br>CGG CTC GAG AAC CTG ATC GCC CAG CTG CCG GGC GAG AAG AAG AAC GGC CTC TTC GGC AAC<br>CTG ATC GCG CTC TCC CTG GGC CTG ACG CCG AAC TTC AAG TCC AAC TTC GAC CTC GCC GAG<br>GAC GCC AAG CTC CAG CTG TCC AAG GAC ACG TAC GAC GAC GAC CTG GAC AAC CTC CTG GCG<br>CAG ATC GGC GAC CAG TAC GCG GAC CTG TTC CTC GCG GCG AAG AAC CTC TCC GAC GCG ATC<br>CTG CTC AGC GAC ATC CTC CGC GTC AAC ACC GAG ATC ACC AAG GCG CCC CTG TCC GCG AGC<br>ATG ATC AAG CGC TAC GAC GAG CAC CAC CAG GAC CTC ACC CTG CTG AAG GCC CTG GTC CGC<br>CAG CAG CTC CCG GAG AAG TAC AAG GAG ATC TTC TTC GAC CAG TCC AAG AAC GGC TAC GCG<br>GGC TAC ATC GAC GGC GGC GCG TCC CAG GAG GAG TTC TAC AAG TTC ATC AAG CCC ATC CTG<br>GAG AAG ATG GAC GGC ACC GAG GAG CTG CTG GTC AAG CTG AAC CGG GAG GAC CTC CTG CGC<br>AAG CAG CGC ACG TTC GAC AAC GGC TCC ATC CCC CAC CAG ATC CAC CTG GGC GAG CTG CAC<br>GCC ATC CTC CGG CGC CAG GAG GAC TTC TAC CCG TTC CTC AAG GAC AAC CGC GAG AAG ATC<br>GAG AAG ATC CTC ACG TTC CGG ATC CCG TAC TAC GTC GGC CCC CTC GCC CGC GGC AAC TCC<br>CGC TTC GCG TGG ATG ACG CGC AAG TCG GAG GAG ACG ATC ACC CCG TGG AAC TTC GAG GAG<br>GTG GTG GAC AAG GGC GCC TCC GCG CAG TCG TTC ATC GAG CGG ATG ACC AAC TTC GAC AAG<br>AAC CTG CCG AAC GAG AAG GTG CTG CCC AAG CAC AGC CTC CTG TAC GAG TAC TTC ACG GTC<br>TAC AAC GAG CTC ACC AAG GTG AAG TAC GTG ACG GAG GGC ATG CGG AAG CCG GCG TTC CTG<br>TCC GGC GAG CAG AAG AAG GCC ATC GTG GAC CTG CTG TTC AAG ACG AAC CGG AAG GTC ACC<br>GTC AAG CAG CTG AAG GAG GAC TAC TTC AAG AAG ATC GAG TGC TTC GAC TCG GTC GAG ATC<br>AGC GGC GTG GAG GAC CGC TTC AAC GCG TCG CTG GGC ACG TAC CAC GAC CTC CTG AAG ATC<br>ATC AAG GAC AAG GAC TTC CTC GAC AAC GAG GAG AAC GAG GAC ATC CTG GAG GAC ATC GTG<br>CTG ACC CTG ACG CTC TTC GAG GAC CGG GAG ATG ATC GAG GAG CGC CTC AAG ACC TAC GCG<br>CAC CTG TTC GAC GAC AAG GTC ATG AAG CAG CTC AAG CGG CGG CGG TAC ACC GGC TGG GGC<br>CGG CTC TCC CGC AAG CTC ATC AAC GGC ATC CGG GAC AAG CAG TCG GGC AAG ACC ATC CTC<br>GAC TTC CTG AAG TCC GAC GGC TTC GCC AAC CGC AAC TTC ATG CAG CTG ATC CAC GAC GAC<br>AGC CTG ACG TTC AAG GAG GAC ATC CAG AAG GCG CAG GTG TCG GGC CAG GGC GAC TCC CTG<br>CAC GAG CAC ATC GCG AAC CTG GCC GGC AGC CCG GCC ATC AAG AAG GGC ATC CTG CAG ACG<br>GTC AAG GTG GTC GAC GAG CTG GTG AAG GTG ATG GGC CGC CAC AAG CCC GAG AAC ATC GTC<br>ATC GAG ATG GCC CGG GAG AAC CAG ACC ACG CAG AAG GGC CAG AAG AAC AGC CGC GAG CGC<br>ATG AAG CGG ATC GAG GAG GGC ATC AAG GAG CTC GGC TCG CAG ATC CTC AAG GAG CAC CCC<br>GTC GAG AAC ACC CAG CTC CAG AAC GAG AAG CTC TAC CTC TAC TAC CTG CAG AAC GGC CGG<br>GAC ATG TAC GTG GAC CAG GAG CTC GAC ATC AAC CGG CTG TCG GAC TAC GAC GTC GAC CAC<br>ATC GTC CCG CAG TCG TTC CTG AAG GAC GAC AGC ATC GAC AAC AAG GTC CTC ACC CGC TCG<br>GAC AAG AAC CGG GGC AAG TCC GAC AAC GTC CCG TCG GAG GAG GTC GTG AAG AAG ATG AAG<br>AAC TAC TGG CGG CAG CTC CTC AAC GCC AAG CTC ATC ACG CAG CGC AAG TTC GAC AAC CTG<br>ACG AAG GCG GAG CGG GGC GGC CTC AGC GAG CTC GAC AAG GCG GGC TTC ATC AAG CGG CAG<br>CTG GTC GAG ACG CGG CAG ATC ACG AAG CAC GTG GCC CAG ATC CTG GAC TCG CGC ATG AAC<br>ACC AAG TAC GAC GAG AAC GAC AAG CTG ATC CGC GAG GTC AAG GTC ATC ACC CTC AAG TCC<br>AAG CTG GTG AGC GAC TTC CGC AAG GAC TTC CAG TTC TAC AAG GTG CGG GAG ATC AAC AAC<br>TAC CAC CAC GCC CAC GAC GCG TAC CTG AAC GCG GTG GTG GGC ACG GCG CTG ATC AAG AAG<br>TAC CCG AAG CTC GAG AGC GAG TTC GTC TAC GGC GAC TAC AAG GTG TAC GAC GTG CGG AAG<br>ATG ATC GCG AAG AGC GAG CAG GAG ATC GGC AAG GCC ACG GCG AAG TAC TTC TTC TAC AGC<br>AAC ATC ATG AAC TTC TTC AAG ACC GAG ATC ACC CTG GCG AAC GGC GAG ATC CGC AAG CGC<br>CCG CTC ATC GAG ACC AAC GGC GAG ACG GGC GAG ATC GTG TGG GAC AAG GGC CGC GAC TTC<br>GCG ACC GTC CGG AAG GTG CTG TCC ATG CCC CAG GTC AAC ATC GTC AAG AAG ACG GAG GTC<br>CAG ACC GGC GGC TTC AGC AAG GAG TCC ATC CTG CCC AAG CGG AAC AGC GAC AAG CTG ATC<br>GCC CGG AAG AAG GAC TGG GAC CCC AAG AAG TAC GGC GGC TTC GAC TCC CCC ACC GTC GCC<br>TAC TCG GTC CTC GTG GTC GCC AAG GTG GAG AAG GGC AAG AGC AAG AAG CTG AAG TCC GTG<br>AAG GAG CTG CTG GGC ATC ACC ATC ATG GAG CGG TCG TCG TTC GAG AAG AAC CCC ATC GAC<br>TTC CTG GAG GCC AAG GGC TAC AAG GAG GTG AAG AAG GAC CTC ATC ATC AAG CTC CCC AAG<br>TAC TCC CTG TTC GAG CTG GAG AAC GGC CGG AAG CGC ATG CTC GCC AGC GCG GGC GAG CTG<br>CAG AAG GGC AAC GAG CTG GCG CTG CCG AGC AAG TAC GTC AAC TTC CTG TAC CTC GCC TCG<br>CAC TAC GAG AAG CTG AAG GGC TCG CCG GAG GAC AAC GAG CAG AAG CAG CTG TTC GTG GAG<br>CAG CAC AAG CAC TAC CTC GAC GAG ATC ATC GAG CAG ATC TCC GAG TTC TCC AAG CGC GTC<br>ATC CTG GCC GAC GCG AAC CTG GAC AAG GTC CTG TCC GCG TAC AAC AAG CAC CGC GAC AAG<br>CCG ATC CGG GAG CAG GCC GAG AAC ATC ATC CAC CTG TTC ACG CTG ACC AAC CTC GGC GCC<br>CCC GCG GCC TTC AAG TAC TTC GAC ACC ACG ATC GAC CGG AAG CGG TAC ACG AGC ACG AAG<br>GAG GTG CTC GAC GCC ACC CTG ATC CAC CAG AGC ATC ACG GGC CTG TAC GAG ACC CGC ATC<br>GAC CTC TCC CAG CTG GGC GGC GAC TGA GTG AGC AAG GGC GAG GAG CTG TTC ACC GGG GTG<br>GTG CCC ATC CTG GTC GAG CTG GAC GGC GAC GTA AAC GGC CAC AAG TTC AGC GTG TCC GGC<br>GAG GGC GAG GGC GAT GCC ACC TAC GGC AAG CTG ACC CTG AAG TTC ATC TGC ACC ACC GGC<br>AAG CTG CCC GTG CCC TGG CCC ACC CTC GTG ACC ACC CTG ACC TAC GGC GTG CAG TGC TTC<br>AGC CGC TAC CCC GAC CAC ATG AAG CAG CAC GAC TTC TTC AAG TCC GCC ATG CCC GAA GGC<br>TAC GTC CAG GAG CGC ACC ATC TTC TTC AAG GAC GAC GGC AAC TAC AAG ACC CGC GCC GAG<br>GTG AAG TTC GAG GGC GAC ACC CTG GTG AAC CGC ATC GAG CTG AAG GGC ATC GAC TTC AAG<br>GAG GAC GGC AAC ATC CTG GGG CAC AAG CTG GAG TAC AAC TAC AAC AGC CAC AAC GTC TAT<br>ATC ATG GCC GAC AAG CAG AAG AAC GGC ATC AAG GTG AAC TTC AAG ATC CGC CAC AAC ATC<br>GAG GAC GGC AGC GTG CAG CTC GCC GAC CAC TAC CAG CAG AAC ACC CCC ATC GGC GAC GGC<br>CCC GTG CTG CTG CCC GAC AAC CAC TAC CTG AGC ACC CAG TCC GCC CTG AGC AAA GAC CCC<br>AAC GAG AAG CGC GAT CAC ATG GTC CTG CTG GAG TTC GTG ACC GCC GCC GGG ATC ACT CTC<br>GGC ATG GAC GAG CTG TAC AAG TAA

Supplementary Table S3: Primer sequence used in DNA template production for all the three templates.

**Primers**	**Sequence**
Forward	TAA TAC GAC TCA CTA TAG GGA CT CAC TAT TTG TTT T
Reverse	TTT TTT TTT TTT TTT TTT TTT TTT TTT TTT TTT TTT TTT TTT TTT TTT TTT TTT TTT TTT ATG CA

Supplementary Table S4: Established RP-HPLC gradient method. The binding corresponds to the mobile phase A composed of 1 X TAE, and the elution corresponds to the mobile phase B composed of 1 X TAE, 25% acetonitrile (v/V). Adapted from [1].

**Time** (min)	**Binding** %	**Elution** %	**Flow** mL.min^{-1}
0	100	0	0.2
1	100	0	0.2
1.5	100	0	0.25
2	94	6	0.35
6	76.5	23.5	0.4
7	0	100	0.4
10	0	100	0.4
11	100	0	0.4
20	100	0	0.4

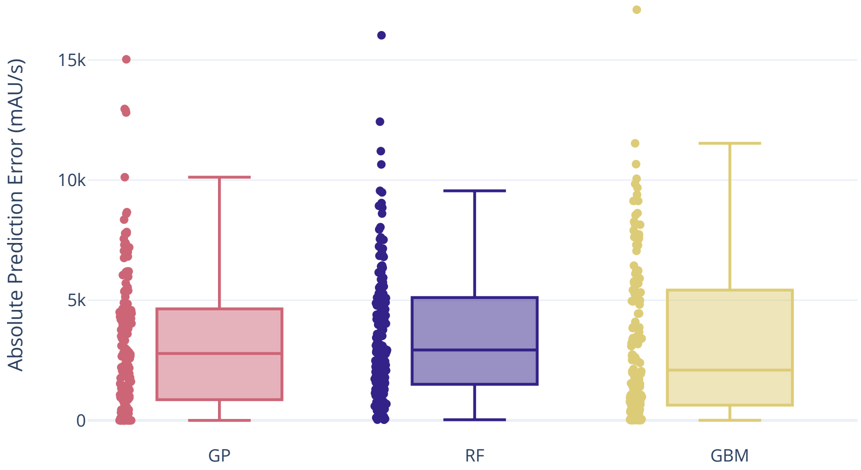

Supplementary Figure S1: Comparison of Gaussian Process (GP), Random Forests (RF) [2] , and Gradient Boosting Machines (GBM)[3] surrogate models using leave-one-out cross validation. The median error for the GP of approximately 3000 mAU.s^{-1} corresponds to 1.8 $g_{mRNA}.L^{-1}$ of error.

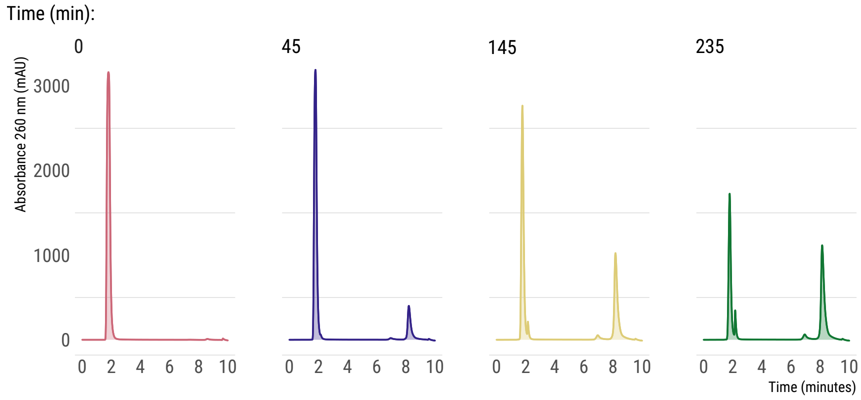

Supplementary Figure S2: Chromatographic profiles of the RP-HPLC runs performed with samples of the parameter setup 5 of the kinetics runs at different time point. The peak at retention time of 2 minutes corresponds to the impurities (NTPs, enzymes), and the peak at 8 minutes corresponds to the mRNA synthesized.

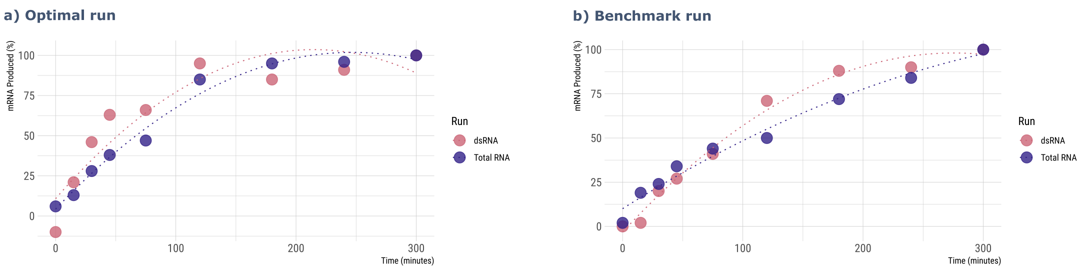

Supplementary Figure S3: Percentage of total mRNA and dsRNA produced in as a function of reaction time considering 100% the highest mRNA concentration produced for each set of runs for both total. a) Total RNA and dsRNA produced using optimal conditions (Run 5). b)Total RNA and dsRNA produced using benchmark conditions [4]. A 2nd order polynomial function was used as a trendline for visualisation purposes. dsmRNA was obtaining by digesting each sample with Rnase T1 and and analysed by HPLC as described in methods.

References

[1] Meredith Packer William Issa. "Methods for hplc analysis". US20210163919A1. 2021.

[2] Leo Breiman. "Random Forests". In: *Machine Learning* 45 (2004), pp. 5–32. DOI: `10.1023/A:1010933404324`.

[3] Jerome H Friedman. "Greedy function approximation: a gradient boosting machine". In: *Annals of statistics* (2001), pp. 1189–1232. DOI: `10.1214/aos/1013203451`.

[4] Stephane Bancel et al. "Manufacturing methods for production of rna transcripts". US Patent App. 14/777,190. Jan. 2016.
